# Frameless robot-assisted stereoelectroencephalography-guided radiofrequency: methodology, results, complications and stereotactic application accuracy in pediatric hypothalamic hamartomas

**DOI:** 10.3389/fneur.2023.1259171

**Published:** 2023-10-19

**Authors:** Ping Li, Yuanfeng Zhou, Qin Zhang, Yuantao Yang, Min Wang, Renqing Zhu, Hao Li, Shuo Gu, Rui Zhao

**Affiliations:** ^1^Department of Neurosurgery, Hainan Women and Children's Medical Center, Haikou, China; ^2^Department of Neurology, Children’s Hospital of Fudan University, National Children’s Medical Center, Shanghai, China; ^3^Department of Neurosurgery, Children’s Hospital of Fudan University, National Children’s Medical Center, Shanghai, China; ^4^Department of Neurosurgery, The First Affiliated Hospital of Hainan Medical University, Haikou, China; ^5^Department of Neurosurgery, Children’s Hospital of Shanghai, Shanghai, China

**Keywords:** stereoelectroencephalography, radiofrequency thermocoagulation, *in vivo* accuracy of electrode implantation, pediatric, hypothalamic hamartoma (HH)

## Abstract

**Objective:**

We aimed to investigate the methodology, results, complications and stereotactic application accuracy of electrode implantation and its explanatory variables in stereoelectroencephalography-guided radiofrequency thermocoagulation (SEEG-RFTC) for pediatric hypothalamic hamartoma.

**Methods:**

Children with hypothalamic hamartoma who underwent robot-assisted SEEG-RFTC between December 2017 and November 2021 were retrospectively analyzed. The methodology, seizure outcome, complications, *in vivo* accuracy of electrode implantation and its explanatory variables were analyzed.

**Results:**

A total of 161 electrodes were implanted in 28 patients with 30 surgeries. Nine electrodes not following the planned trajectories due to intraoperative replanning were excluded, and the entry point and target point errors of 152 electrodes were statistically analyzed. The median entry point error was 0.87 mm (interquartile range, 0.50–1.41 mm), and the median target point error was 2.74 mm (interquartile range, 2.01–3.63 mm). Multifactor analysis showed that whether the electrode was bent (b = 2.16, *p* < 0.001), the length of the intracranial electrode (b = 0.02, *p* = 0.049), and the entry point error (b = 0.337, *p* = 0.017) had statistically significant effects on the target error. During follow-up (mean duration 31 months), 27 of 30 (90%) procedures were seizure-free. The implantation-related complication rate was 2.6% (4/152), and the major complication rate in all procedures was 6.7% (2/30).

**Conclusion:**

Robot-assisted SEEG-RFTC is a safe, effective and accurate procedure for pediatric hypothalamic hamartoma. Explanatory variables significantly associated with the target point localization error at multivariate analysis include whether the intracranial electrode is bent, the intracranial electrode length and the entry point error.

## 1. Introduction

Hypothalamic hamartoma (HH) is a rare congenital developmental abnormality of brain tissue, with an incidence of about 1/50,000 to 1/100,000 (1). HHs are more common in children and are caused by a well-recognized pathology that may develop into gelastic seizures, central precocious puberty (CPP) and even cognitive and behavioral problems (2–4). The cellular mechanisms responsible for intrinsic epileptogenesis of HH tissue are not fully understood but some studies showed that neuronal gap junctions between small GABAergic HH neurons participate in the genesis of epileptic-like discharges (5, 6). Open surgery is the traditional treatment modality of HH, and is associated with favorable prognosis. However, the operation was complicated and the major complication rate reported was high, including thalamic infarction, memory impairment, postoperative central diabetes insipidus, and hyperphagia (2, 7–10). In recent years, with emerging technologies applied in neurosurgery, more institutions have adopted stereotactic surgery, such as stereoelectroencephalography-guided radiofrequency thermocoagulation (SEEG-RFTC) or magnetic resonance imaging (MRI)-guided laser interstitial thermal therapy (LITT), for the treatment of HH (11–13).

SEEG was first developed by Talairach and Bancaud in the 1950s (14–16). As they described, the SEEG electrode was implanted into the patient’s brain with the assistance of a stereotactic graph (such as a Leksell head frame) based on the imaging results. Previous studies have shown that this method is accurate and safe (17). The introduction of high-resolution imaging, robotic stereotactic systems and RFTC greatly contributed to the technical evolution of this methodology in the past 20 years (11, 14, 18–21). Now, the SEEG electrode can be connected to heat the contacts inside the lesion, which can thermocoagulate and damage the diseased tissue and achieve the same effect as craniotomy in selected cases (11, 22).

The treatment of HH with SEEG-RFTC has the advantages of simple operation, less trauma, fewer complications and a high postoperative seizure remission rate, and is therefore widely applied (22–24). Theoretically, the surgical effect and complications are related to the accuracy of intraoperative electrode implantation. Deviation of the electrode insertion trajectory from the planned trajectory may result in damage to vessels and tissues and hence major complications (25), whereas deviation of the electrode tip position from the planned target position leads to incomplete lesion tissue ablation and unsatisfactory postoperative epilepsy relief effects. There are some articles on the *in vivo* accuracy of SEEG for presurgical investigation (17–20, 26–29), but to the best of our knowledge, no studies of the accuracy of SEEG for treatment of pediatric HH have been published. Compared with adults, children have a thinner and more fragile skull, and young children have patent skull sutures, which may affect the accuracy of electrode implantation (26, 28). In HH patients, the lesions are deep and adjacent to important structures, requiring longer intracranial electrodes and higher accuracy, rendering SEEG for pediatric HH unique. In this study we investigated the accuracy of electrode implantation and its explanatory variables in SEEG-RFTC for pediatric HH. Our findings also have important clinical implications for LITT.

## 2. Methods

### 2.1. Patient selection

All HH patients who were treated with robot-assisted SEEG-RFTC in the Children’s Hospital of Fudan University (Shanghai, China) between December 2017 and November 2021 were eligible.

### 2.2. Preoperative preparation

All pediatric patients were imaged by high-resolution MRI scans, including 3.0 T MRI with three-dimensional (3D) T1-weighted sequence (1 mm, including no-contrast and double-contrast sequences) and 3D coronal FLAIR sequence (1 mm) with fat suppression.

Stereotactic trajectories were planned using SINO PLAN (Sino-precision, Beijing, China). We adopted a high-density focal stereo-array electrode implantation strategy (30) (stereotactic electroencephalography guided bipolar coagulations were performed between two contiguous contacts of the same electrode, or between two adjacent contacts of different electrodes) and mainly based on the following principles for trajectory planning: (1) for small HHs, the targets were in the center of the lesion to achieve complete ablation, with 3–5 electrodes per implantation; (2) for large HHs, for which it is difficult to achieve complete ablation, the targets were at the junction between the lesion and the hypothalamus to achieve complete disconnection, with 6–10 electrodes per implantation; and (3) for the trajectory of each electrode, vessels in MR sequences were avoided with a safety margin of 2 mm. In order to improve the stability of screw, we preferred the parietal bone as the entry point, orthogonal implantation, and the implantation angle perpendicular to the skull section. However, due to high-density focal stereo-array electrode implantation strategy, some electrodes did not meet the above conditions.

### 2.3. Electrode implantation

**Figure 1:**
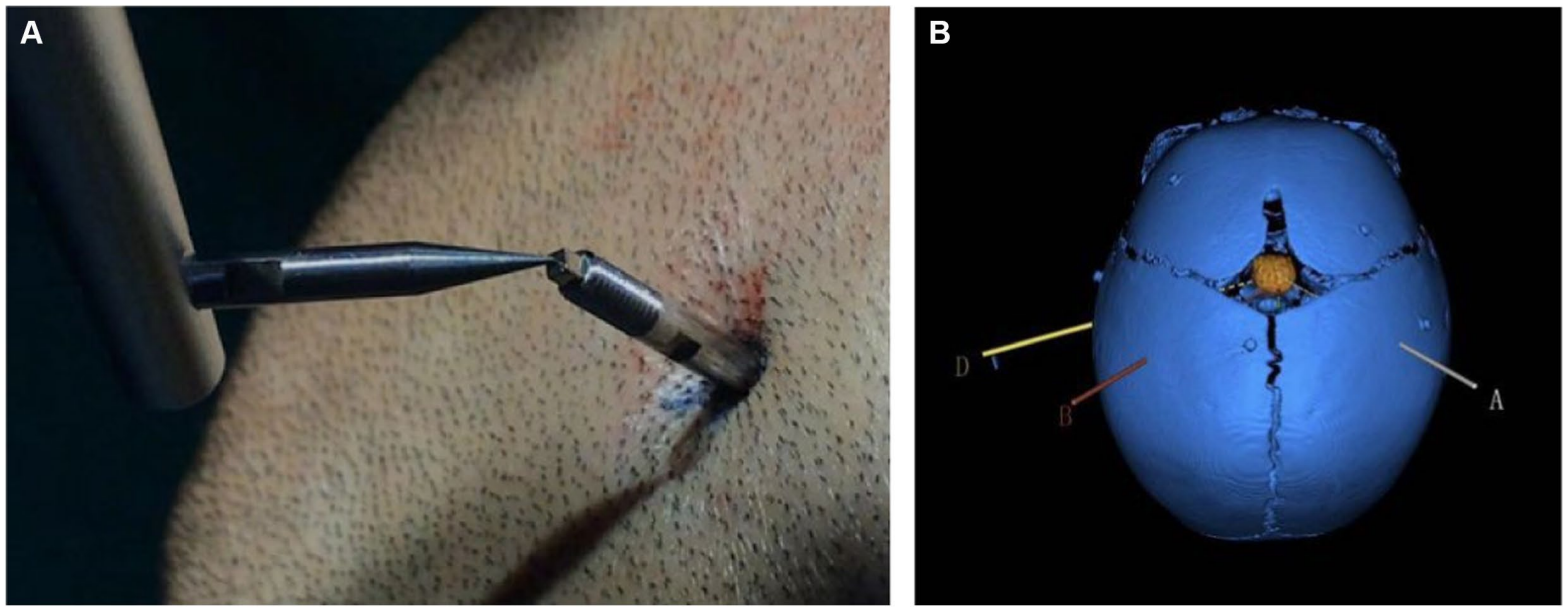
**(A)** Co-registration was performed by a robotic probe point-to-point contacting the markers fixed into the pediatric skull. **(B)** Schematic diagram of an electrode trajectory and an HH lesion after reconstruction. The colored line is the planned electrode trajectory path, the orange intracranial mass is the HH lesion after reconstruction, and the blue skull is the skull of the child after 3D reconstruction. The patient was 8 months old and had patent skull sutures.

All HH patients with seizures underwent SEEG exploration using a frameless robot-assisted SEEG system following a previously described protocol with modifications (31). According to our SEEG recording, all gelastic seizures were correlated with discharges within the hypothalamic hamarthomas (HH), whereas other seizure types were related to discharges affecting cortical regions, which sometimes seemed to be triggered by HH. And almost all other seizure types run down after the thermal coagulation of HH, it suggests that there may be a “kindling-like “relationship between the HH and the neocortex or widespread epileptogenesis (32). Therefore, in our study HH was determined to be the sole Seizure Onset Zone (SOZ) in all patients and we implanted only electrode in the HH region. The Leksell frame was only used as the fixation system to allow better registration and to avoid possible collisions with the robotic arm. For registration, we used a conventional method of placing the tip of the registration probe in a position tangent to the groove of the bone screws (Figure 1). Only registrations with small errors (<0.5 mm) were accepted and used for the implantation. The robotic arm reached the designated position according to preoperative planning automatically. For each electrode, we incised the skin, drilled in the bone and burned the dura with an electrocoagulation needle. A guidance screw was inserted into the skull and a stylet was introduced to initiate the electrode trajectory and measure the depth of the target. After determining the suitable contact electrode according to the target depth, the electrodes (0.8 mm in diameter, ALICIS, Besançon, France; 0.8 mm in diameter, Sino-precision) were implanted through a guidance screw and then secured to the screw at its final position.

### 2.4. RFTC

Once all electrodes were inserted, in order to rule out intraoperative complications and verify the exact contact locations, postoperative 3D computed tomography (CT) co-registered with preoperative 3D T1-weighted MRI was performed. Afterwards, the patients were transferred to the Pediatric Neurology Unit for continuous video-SEEG monitoring. After recording the ictal/interictal activities and seizure foci mapping, contacts within the HH were selected for RFTC. The coagulation power was increased to 3.5 W within 15 s and maintained at 3.5 W for 45 s with a 1-min interval. The thermocoagulation strategy included thermocoagulation between two adjacent contacts of the same electrode and cross-thermocoagulation between different electrodes (interval ≤ 8 mm).

### 2.5. Application accuracy

**Figure 2:**
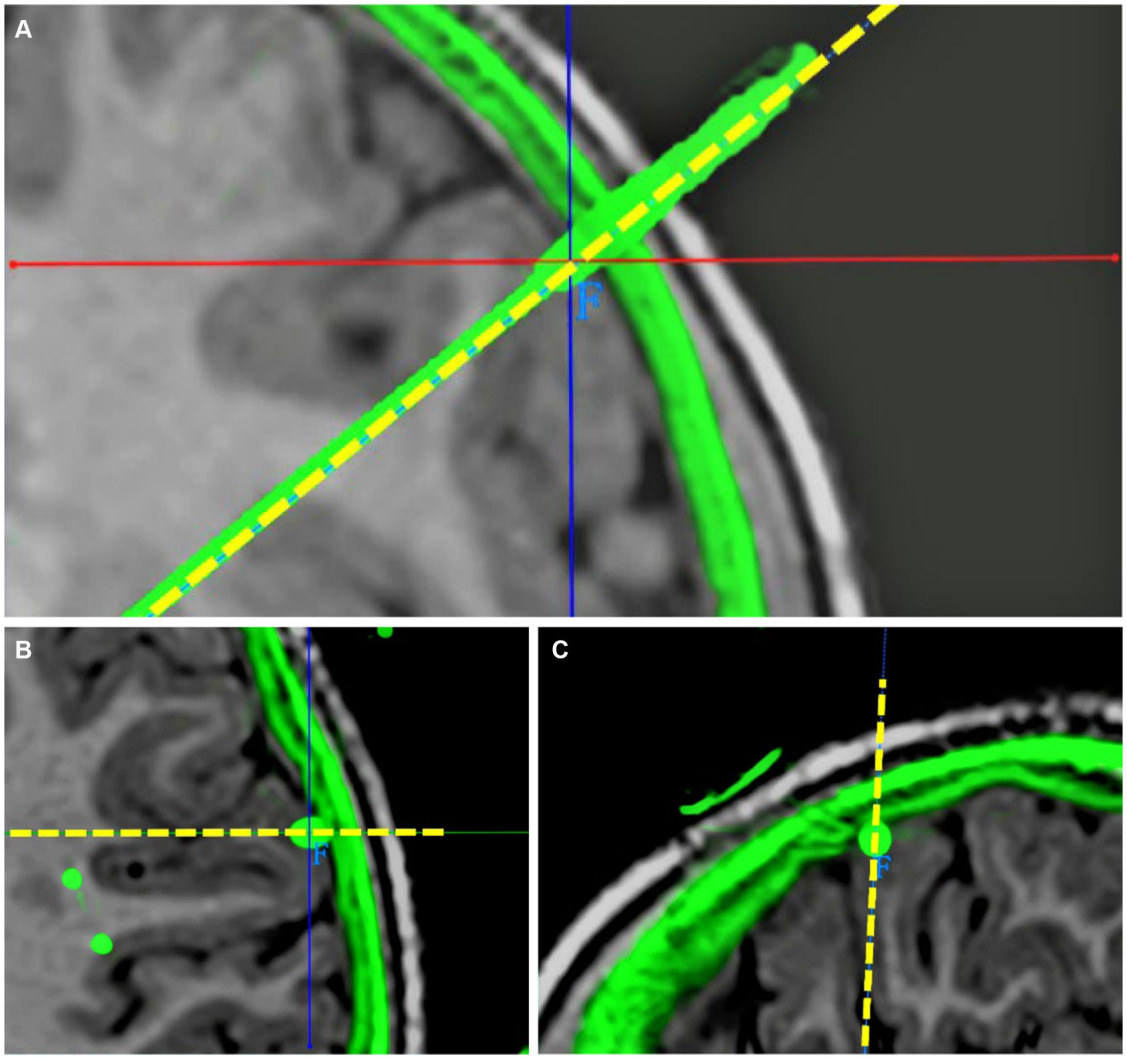
Determination of EPLE. **(A)** Coronal visualization of planned and actual electrode trajectories. **(B)** The planned and actual electrode tracks are displayed in the transverse plane. Their entry points coincided. **(C)** The planned and actual electrode tracks are shown in the sagittal plane. Their entry points coincided. The junction of solid red and blue lines is the center of the actual electrode track entry point, the dotted yellow line is the planned electrode track, and the green part outside the cerebral cortex is the skull shadow in the postoperative CT after registration. The EPLE is 0.31 mm.

**Figure 3:**
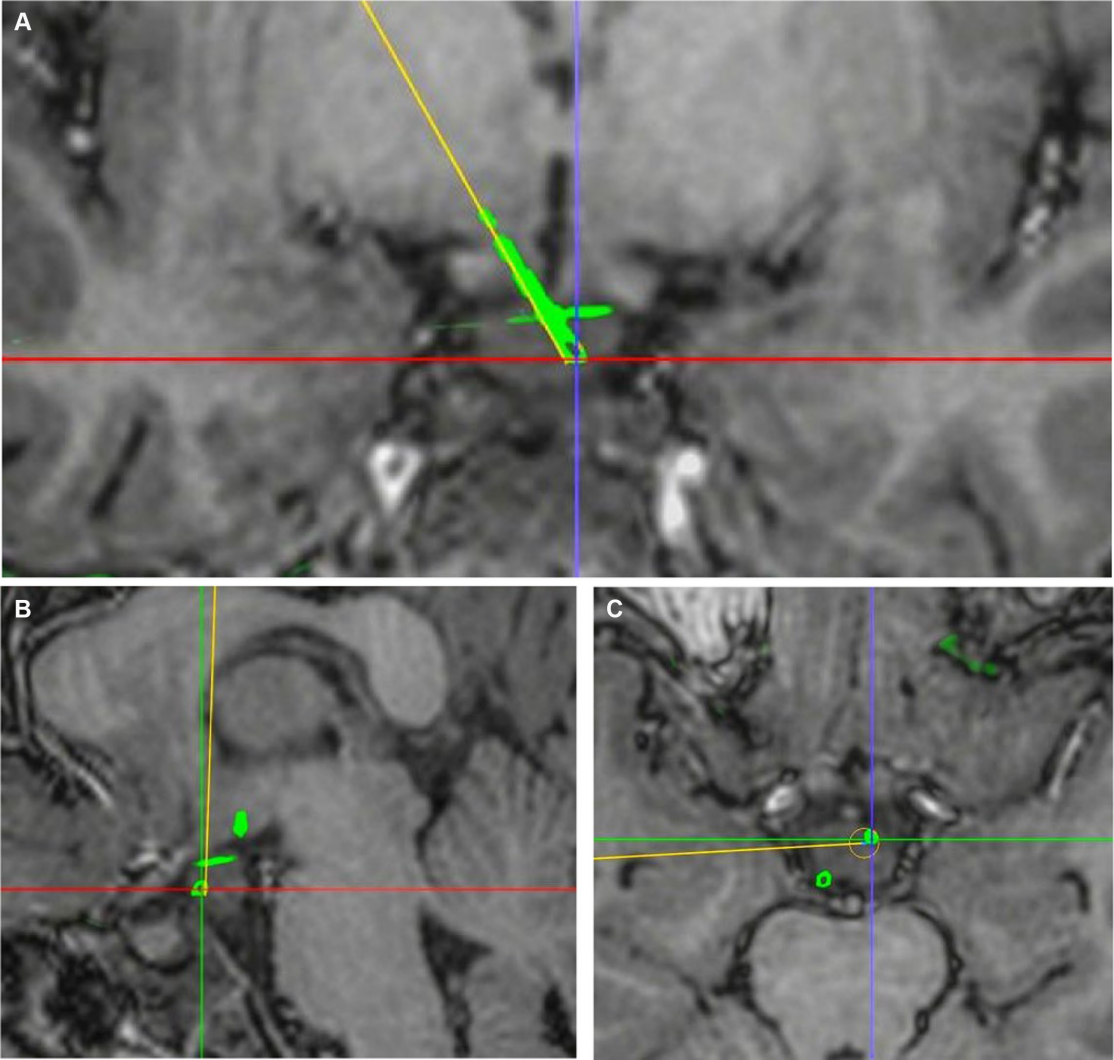
Determination of TPLE. **(A)** Coronal visualization of planned and actual electrode trajectories. **(B)** The planned and actual electrode tracks are displayed in the sagittal plane. **(C)** The planned and actual electrode tracks are shown in the transverse plane. The junction of red and purple lines is the center of the actual electrode trajectory target, and the yellow line is the planned electrode trajectory. The TPLE is 1.08 mm.

The exact contact locations of implanted electrodes were verified using postoperative 3D CT co-registered with preoperative 3D T1-weighted MRI. The preoperative planning trajectory imaging data in the robot system were integrated with the postsurgical CT. The entry point was defined as the center position where the electrode trajectory entered the cortex, and the target was defined as the final position of the electrode tip. The planned and the actual trajectory were determined from the coronal, sagittal and transverse planes. The unsigned Euclidean distance between the planned and the actual entry point (entry point localization error [EPLE]) and between the planned and the actual target point (target point localization error [TPLE]) were measured using the distance measurement function in the robot software system (Figures 2, 3).

### 2.6. Statistical analysis

Statistical analysis was performed using IBM SPSS Statistics version 26 (IBM SPSS Inc., Chicago, United States). In the sub-dataset of 152 electrodes implanted in HH children, several explanatory variables (listed in Tables 1, 2) were considered to investigate potential sources of errors and their possible association with TPLE.

**Table 1:** Bivariate analysis of numerical variables and TPLE.^a^

Variable	Median (IQR)	TPLE	
		*r*	*p*
Age(years)	3.80 (1.50–6.10)	0.086	0.291
Skull thickness(mm)	3.11 (2.22–3.71)	−0.024	0.772
Soft tissue thickness(mm)	5.13 (4.32–6.57)	−0.004	0.959
Acute skull angle(degrees)	75 (72–79)	0.202	0.013^b^
Intracranial length(mm)	83.94 (75.61–90.19)	0.151	0.064^b^
Electrode-ventricle distance(mm)	3.05 (4.87–8.48)	0.126	0.123
EPLE (mm)	0.87 (0.50–1.41)	0.248	0.002^b^

a TPLE, target point localization error; EPLE, entry point localization error; IQR, interquartile range. Soft tissue thickness is the distance between the external edge of the skin and the external edge of the skull. Acute skull angle is the acute angle between the planned trajectory and the sliced face of the skull. Intracranial length is the distance between entry and target points. Electrode–ventricle distance is the closest proximity of the planned trajectory to the lateral ventricles. None of the values are normally distributed (Shapiro–Wilk normality test *p*-values are < 0.05 for all variables). Bivariate analysis (Spearman correlation) was performed.

b Significant.

**Table 2:** Bivariate analysis of categorical variables and TPLE.^a^

Variable	Categories	Frequency (%)	TPLE	
			z/h	*p*
Skull	Frontal bone	10(6.6)	0.40^c^	0.818
	Parietal bone	110 (72.4)		
	Temporal bone	32 (21.1)		
Orthogonal/Oblique	Orthogonal	73 (48)	−1.20^b^	0.231
	Oblique	79 (52)		
Electrode type	Alcis	112 (74)	−0.02^b^	0.983
	Sino-precision	40 (26)		
Bending	No	142 (93.4)	−4.40^b^	0.000^d^
	Yes	10 (6.6)		
Cerebrospinal fluid	No	110 (72.4)	−0.46^b^	0.644
	Yes	42 (27.6)		
Tissue	Nuclei	98 (64.5)	1.16^c^	0.764
	Internal capsule	38(25)		
	Lobe	14(9.2)		
	Ventricle	2 (1.3)		
Electrode number	3	13 (8.5)	12.50^c^	0.029^d^
	4	20 (13.1)		
	5	32 (21.1)		
	6	34 (22.4)		
	7	34 (22.4)		
	10	19 (12.5)		

a TPLE, target point localization error; the planned trajectories orthogonal to the sagittal plane are divided into orthogonal groups, others are divided into oblique groups. Cerebrospinal fluid is positive when the planned trajectory passes through the cerebrospinal fluid in the ventricles or cisterns. Electrodes were classified according to the passed special brain tissues such as nuclei, internal capsule and ventricle; trajectories without special tissues were thought to have passed only through the brain lobe.

b Mann–Whitney U-test.

c Kruskal–Wallis H-test.

d Significant.

For bivariate analysis, the Spearman correlation test was used to analyze numerical variables, and the Kruskal–Wallis rank sum test was performed to analyze categorical (binomial or multinomial) variables. To include any significant variable or confounder for multivariable analyses, variables with univariable *p* < 0.10 were selected to build a multivariate model. Multivariate analysis of the included variables was performed using a generalized linear model. Unless stated otherwise, a significance level of *p* < 0.05 was used. All bivariate tests should be understood as constituting exploratory data analysis; no adjustments for multiple testing were made.

## 3. Results

### 3.1. Patient demographics

Between December 2017 and November 2021, a total of 450 children with refractory epilepsy were operated on in our hospital, of which 35 HH pediatric patients underwent 37 robotic-assisted SEEG-RFTC surgeries, and HH patients account for about 0.6% of neurosurgery inpatients in our hospital. Seven children with lost preoperative trajectory planning data were excluded, and a total of 161 electrodes were implanted in 30 surgeries (28 children). Two patients underwent surgery twice in our hospital. The demographics are shown in Table 3. In total, 15 patients were male and 13 were female, and the median age at surgery was 3.8 years (range 0.6–12.0 years). The median course of disease was 1.0 year (range 0.1–11.0 years). Informed consent was obtained from their guardians.

**Table 3:** Characteristics of 30 patients with HH who underwent SEEG-RFTC.^a^

Patient Characteristics	Total cohort (*n* = 30)	%
Gender		
Male	15	50.0
Female	15	50.0
Median age at RFTC, years (range)	3.8 (0.6–12.0)	NA
Median course of disease, years(range)	1.0 (0.1–11.0)	NA
Classification (Delalande)		
I	9	30.0
II	6	20.0
III	10	33.3
IV	5	16.7
Largest diameter of HH, mm (range)	17.83 (12.03–46.45)	NA
Volume of HH, cm^3^ (range)	1.83 (0.27–33.15)	NA
CPP	16	53.3
Developmental delay	14	46.7
Previous surgery	5	16.7
Accompanied abnormality		
Arachnoid cyst	1	3.3
Polydactyly	2	6.7
No. of implanted electrodes		
3–5	17	56.7
6–10	13	43.3
Seizure outcome^b^		
1	27	90
2–4	1	3.3
5–6	2	6.7

a SEEG-RFTC, stereoelectroencephalography-guided radiofrequency thermocoagulation; CPP, central precocious puberty; NA, not applicable.

b According to the ILAE operational classification system of seizure types of 2017. ILAE, International League Against Epilepsy.

In all 30 procedures, patients were classified according to the Delalande classification system as type I (*n* = 9), type II (*n* = 6), type III (*n* = 10), or type IV (*n* = 5). The median largest HH diameter was 17.83 mm (range 12.03–46.45 mm), and the median HH volume was 1.83 cm^3^ (range 0.27–33.15 cm^3^). All patients presented with seizures, 16 of which had CPP, and 14 of which had developmental delay. Patients with small HH typically presented with late-onset seizures characterised only by gelastic seizures and usually absented cognitive/behavioral impairment. Three patients presented with other abnormalities: arachnoid cyst (*n* = 1) and polydactyly (*n* = 2). Five patients had a history of surgery: two cases had received open surgery only, one had received robot-assisted SEEG-RFTC only, and two patients had undergone both.

### 3.2. Implantation characteristics

In our study, the electrodes were all implanted in HH. A total of 161 electrodes were implanted in 28 patients with 30 surgeries, with 3–10 electrodes per implantation (3 electrodes *n* = 5, 4 electrodes *n* = 5, 5 electrodes *n* = 7, 6 electrodes *n* = 6, 7 electrodes *n* = 5, 10 electrodes *n* = 2). Nine electrodes for which the planned trajectories were not followed due to intraoperative replanning were excluded, and the entry point and target point errors of 152 electrodes were statistically analyzed. Analysis of complications was performed for all 161 electrodes.

Due to the deep location of the HH, the intracranial electrodes are longer than for most cortical targets. The median intracranial length was 83.94 mm [interquartile range (IQR) 75.61–90.19 mm]. Electrodes were classified according to passed brain tissues such as nuclei (*n* = 98), internal capsule (*n* = 38), and ventricle (*n* = 2). Trajectories without special tissues were thought to have passed only through the brain lobe (*n* = 14). Ten electrodes were bent inside the skull. We used two types of electrodes from different companies: Alcis (*n* = 112) and Sino-precision (*n* = 40).

We adopted a high-density focal stereo-array electrode implantation strategy to expand the scope of damage. There are about 20 contacts were coagulated in small lesions and more than 70 contacts in large lesions.

### 3.3. SEEG findings and seizure outcomes

All the patients had at least once scalp video-electroencephalogram monitoring before surgery and SEEG recording after the implantation. Interictal spikes and spontaneous seizures were recorded from the hamartoma in all patients presenting with gelastic seizures. Focal or multifocal of the interictal epileptiform discharges were seen in all cases and nearly one-third of ictal epileptiform abnormalities presented evolving generalized fast rhythmic activity.

All patients had an in-clinic follow-up assessment at least 12 months after RFTC according to the International League Against Epilepsy classification. The mean follow-up duration was 31.40 ± 9.75 months. Of 30 procedures, a class I outcome was achieved in 27 cases (90%). The outcomes of the other three surgeries were classified as class IV, class V, and class VI (*n* = 1 each). The patients classified as class V or VI required two surgeries and were free of gelastic seizures at follow-up after more than 12 months.

### 3.4. Complications

**Figure 4:**
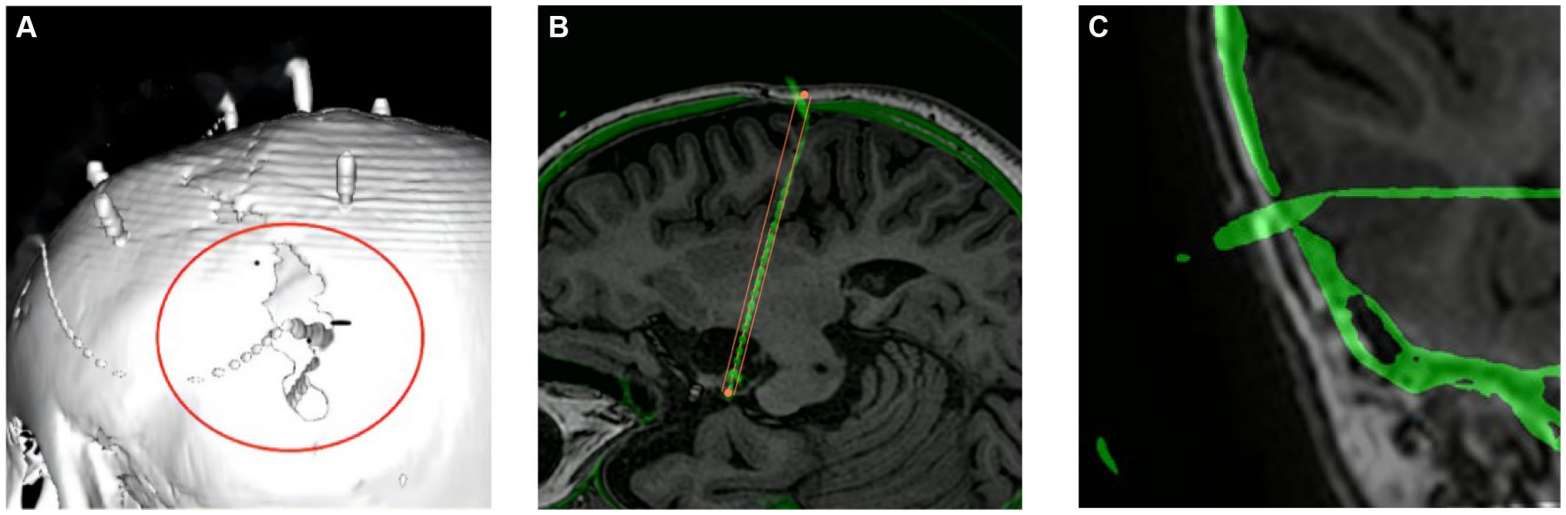
Screw dislocation. Panels **(A,B)** show the same displaced bolt at a weak point in the skull after a previous craniotomy. **(C)** The displaced bolt in temporal bone.

Complications are detailed in Table 4. All patients tolerated the procedure well. On routine postimplantation CT, two patients with small-volume bleedings (one subdural hematoma and one intraparenchymal hematoma) were asymptomatic, and none required specific surgical interventions. In two patients, there was a malposition of one fixation screw through the tabula externa and interna into the cranial cavity (Figure 4). Because the patient was asymptomatic and target location was still within the lesion, the screw was left in position. The implantation-related complication rate was 2.6% (4/152).

**Table 4:** Complications.^a^

Complication	Classification	Total, *n*	%
associated with implantation			
Bleeding	Minor	2/152	1.3
Screw malposition	Minor	2/152	1.3
associated with thermocoagulation			
Electrolyte abnormalities	Minor	3/30	10
Oculomotor palsy	Minor	1/30	3.3
Hypothyroidism	Major	1/30	3.3
Short-term memory impairment	Major	1/30	3.3

a Complications were considered major when requiring surgical treatment or resulting in a permanent neurological deficit.

Several complications were caused by thermocoagulation, three patients developed electrolyte abnormalities and another suffered from oculomotor palsy manifested as right eyelid droops. Conservative treatment was effective and all patients showed complete remission of clinical symptoms. Hence, they were considered minor complications. A complication causing a permanent neurological deficit was considered major. Two patients showed major complications. One patient with hypothyroidism required long-term Levothyroxine treatment, and another suffered from short-term memory impairment. The major complication rate in all procedures was 6.7% (2/30).

### 3.5. Application accuracy

#### 3.5.1. Euclidean distance

The *in vivo* application accuracy was analyzed. The median EPLE was 0.87 mm (IQR, 0.50–1.41 mm) and the median TPLE was 2.74 mm (IQR, 2.01–3.63 mm). The maximum EPLE was 2.83 mm, and the minimum error was 0 mm. The maximum TPLE was 8.56 mm, and the minimum error was 0 mm. In Table 5, our values are compared with those of other reports of similar measurements (*in vivo* Euclidean errors for implantation of stereotactic devices).

**Table 5:** Application accuracy: literature review.^a^

Reference (Period)	Population	Methods	Co-registration	Electrodes	Intracranial length, mm	EPLE, mm	TPLE, mm
Cardinale et al. (27), (1996–2011)	Children, Adults	Framebase Talairach	/	517	NA	1.43 (0.91–2.21)^b^	2.69 (1.89–3.67)
		Robot Neuromate	Bone fiducial	1,050	46.2 (35.9–52.9)	0.78 (0.49–1.08)^b^	1.77 (1.25–2.51)
Nowell et al. (33), (NA)	Children, Adults	Frameless Medtronic Stealth Vertek	Surface fiducial	187	NA	NA	3.45 (IQR 3.6)
González et al. (20), (2009–2013)	Children, Adults	Robot ROSA	Surface fiducial	500	NA	1.20 (0.78–1.83)	1.70 (1.20–2.30)
Ollivier et al. (34), (2010–2016)	Children, Adults	Robot ROSA	Surface fiducial	857	NA	1.10 (0.15–2.48)	2.09 (1.06–3.72)
Van der Loo et al. (29), (2008–2016)	Children, Adults	Framebase Talairach	/	866	41.0 (30.7–55.5)	1.54 (0.92–2.28)	2.93 (1.98–4.20)
Candela-Cantó et al. (26), (2016–2018)	Children	Robot Neuromate	Surface fiducial	164	NA	1.57 (1.00–2.25)	1.77 (1.20–2.60)
Sharma et al. (28), (2014–2017)	Children	Frameless Medtronic Stealth Vertek	Surface fiducial	218	NA	5.5 (4.0–6.4)	4.5 (2.8–6.1)
		Robot Neuromate	Surface fiducial		NA	0.71(0.47–1.03)	1.07 (0.71–1.59)
Bourdillon et al. (17), (2012–2017)	Children, Adults	Framebase Talairach	/	628	NA	NA	4.00 ± 0.97^c^
		Robot Neuromate	NA	565	NA	NA	1.15 ± 0.36^c^
Present study (2017–2021)	Children	Robot Sino-precision	Bone fiducial	152	83.94 (75.61–90.19)	0.87 (0.50–1.41)	2.74 (2.01–3.63)

a EPLE, entry point localization error (defined as the unsigned Euclidean distance between planned entry coordinates and the post-implantation positions of the electrode entry point); TPLE, target point localization error (defined as the unsigned Euclidean distance between planned tip positions and the position of the tip of the implanted electrode); NA, not applicable.

b The EPLE is defined as the unsigned Euclidean distance between planned entry points and the major axis of the implanted electrode.

c Mean ± SD; SD, standard deviation.

#### 3.5.2. Explanatory variables

Explanatory variables significantly associated with TPLE in univariate analysis were the angle between the electrode long axis and the skull section (*r* = 0.202, *p* = 0.013), the length of the intracranial path (*r* = 0.151, *p* = 0.064), the occurrence of intracranial deviation of the electrode (*z* = 4.40, *p* < 0.001), the total intracranial electrodes (h = 12.98, *p* = 0.024) and EPLE (*r* = 0.248, *p* = 0.002) (Tables 1, 2).

Explanatory variables significantly associated with TPLE in multivariate analysis were EPLE (b = 0.337, *p* = 0.017), the occurrence of intracranial deviation of the electrode (b = 2.16, *p* < 0.001) and the length of the intracranial path (b = 0.02, *p* = 0.049) (Table 6).

**Table 6:** Multivariate analysis.^a^

Variable	Categories	B	B value	Wald	95%CI Wald	*p*
Intercept		−0.58	1.21	0.23	−2.95-1.78	0.628
Bending	No^b^					0.000^c^
	Yes	2.16	0.38	32.80	1.42–2.90	
Electrode number	3^b^					0.107
	4	0.88	0.40	4.88	0.10–1.66	
	5	0.66	0.37	3.25	−0.06-1.38	
	6	0.41	0.36	1.25	−0.31-1.12	
	7	0.90	0.37	5.96	0.18–1.61	
	10	0.84	0.41	4.30	0.05–1.64	
Acute skull angle(degrees)		0.01	0.02	0.30	−0.03-0.05	0.582
Intracranial length(mm)		0.02	0.01	3.89	0.00–0.04	0.049^c^
EPLE		0.337	0.14	5.65	0.06–0.62	0.017^c^

a EPLE, entry point localization error.

b Reference category.

c Significant.

## 4. Discussion

Children have a thinner and more fragile skull, and young children have patent skull sutures, which may affect the accuracy of electrode implantation. For HH patients, the lesions are deep and adjacent to important structures. Therefore, SEEG for pediatric HH may represent a special entity. In this study we investigated the accuracy of electrode implantation and its explanatory variables in SEEG-RFTC for pediatric HH.

### 4.1. Accuracy

Our entry point accuracy measurements are in agreement with those obtained by other groups using a robot (Table 5). The median Euclidean entry error in our study was 0.87 mm. Cardinale et al. (27) reported a median error of 0.78 mm. Similarly, González et al. (20) reported a median EPLE of 1.20 mm. The *in vivo* accuracy at entry reported for robot-assisted SEEG implantations is superior to that of other methods. Cardinale et al. (27) compared the accuracy of their reported data with their previous Talairach framework and reported that the median EPLE (the distance of the planned entry point to the long axis of the actual trajectory) in the Talairach frame system was 1.43 mm and in the robot navigation system it was 0.78 mm. These results are similar to the findings of Sharma et al., who reported a median accuracy at the entry of 0.71 mm for the robot system and 5.50 mm for the frameless system (28).

The EPLE was not significantly larger because the participants were children, the skull was thin, and the cranial sutures were patent, which was consistent with the results of two other studies (26, 28) in children (Table 5). However, no multivariate studies of EPLE were conducted here. Some studies (26, 35) proposed that the use of SEEG in children with a skull thickness of <2 mm and younger than 2 years of age should be restricted, but no significant difference in skull thickness and age was found in the multivariate analysis of TPLE in our study. But then, in this study there were indeed two cases of screw dislocation (Figure 4). One screw was located at a weak point in the skull after previous craniotomy, and in the other case an electrode was located in the temporal region, possibly due to temporal muscle contraction. Since the bolt is bent after implantation, it has little effect on the target. Candela-Canto et al. (26) also found an electrode implanted in the temporal part of a 7-year-old child with an oblique trajectory and a bone thickness of 3 mm, in which the bolt was shifted due to temporal muscle contraction. Children have a thinner and softer skull than adults, and infants may have their skull joints and fontanelle not yet closed. These factors all increase the instability of the bolt (28, 36). In particular, young children, who need to be monitored in the ward after electrode implantation, inevitably touch the exposed bolts, so there is a need for higher bolt stability. More studies are needed to determine whether the use of SEEG electrodes in thin skulls, young patients, and temporal lobes should be restricted. In other words, we should pay more attention to skull weakness and the temporal lobe when designing SEEG trajectories (28).

Regarding TPLE, our values are greater than those reported by other institutional robots, similar to frame-based values, and smaller than values obtained using frameless systems. We reported a median target point error of 2.74 mm. Van der Loo et al. (29) reported the largest SEEG implantation error of the Talairach framework system to date. Specifically, 866 electrodes were implanted in 76 operations, and the median Euclidean distance target error was 2.93 mm. Other institutions using the Talairach framework system reported TPLE values of 1.4–4.0 mm (17, 27). The median TPLE reported by other institutions using a robot system is 1.07–2.09 mm (17, 20, 26–28, 34). We reported a median error at the target of 2.7 mm, which is slightly larger than those in most published series. The underlying reason is unclear. It may be due to (i) the fact that the electrodes in our study were significantly longer than those of other studies of only preoperative evaluation of SEEG monitoring (83.94 mm vs. 41.0 mm) (27, 29) or (ii) the age of patients which median is 3.8 years, the skull is thinner than in adults, and some children have patent cranial sutures.

**Figure 5:**
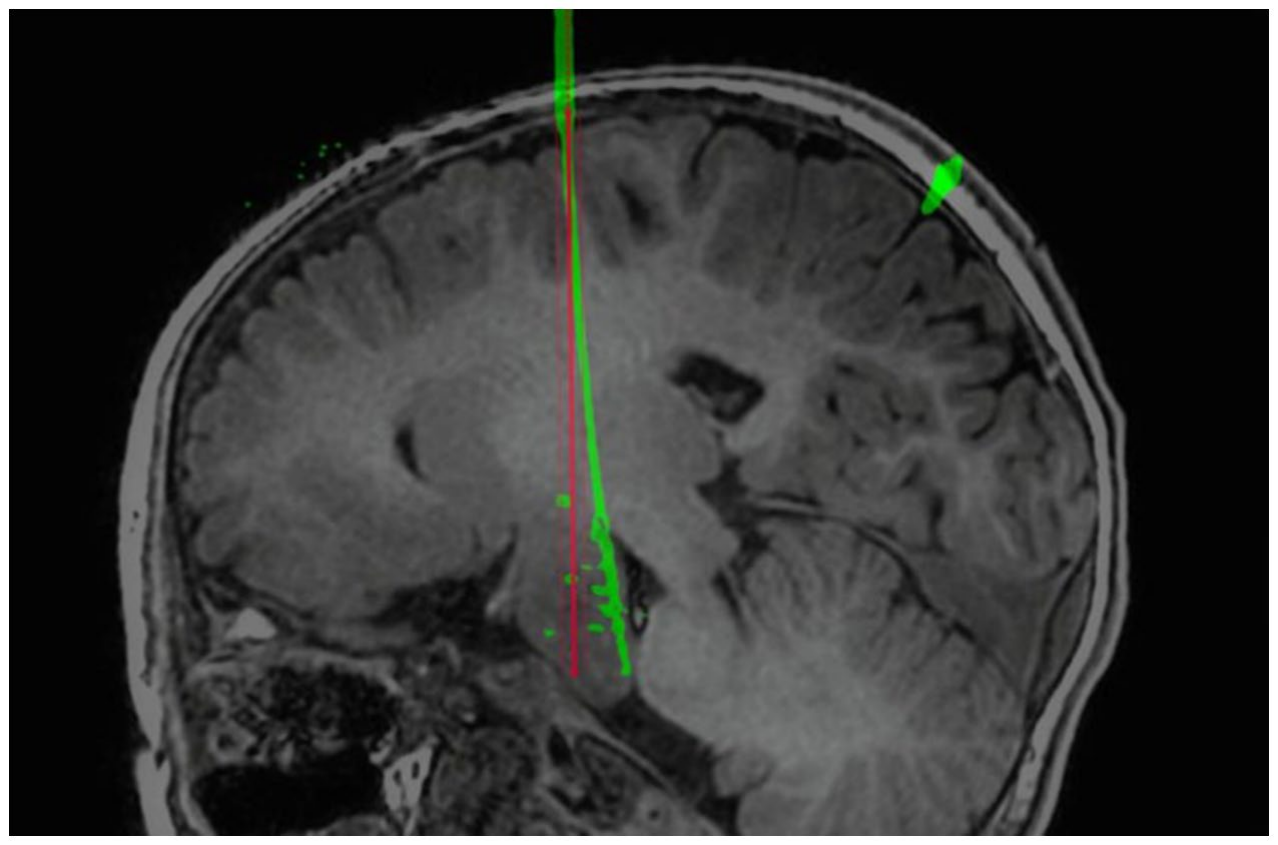
Example of self-bending of an intracranial electrode. The electrode spontaneously bent in the brain. The red line is the planned electrode track, and the green line is the actual electrode track in postoperative CT after reconstruction.

In our multivariate analysis of TPLE, we did find a clear positive correlation between intracranial electrode length, EPLE, and TPLE. This is similar to findings reported by other institutions (27–29, 37). However, van der Loo et al. (29) found that the length of the electrode was not significantly associated with the results and that short electrodes could also show bending immediately after entering the skull, resulting in a large deviation. Indeed, we found that whether or not intracranial electrodes are bent is also significantly related to TPLE. Although the number of electrodes that bend is small, electrode bending often leads to large errors (Figure 5) (27). We performed univariate analysis of factors that may influence electrode bending, but found no statistically significant variables (not listed here). Whether electrode bending is related to factors such as the type of electrode, the structure passed through the skull, etc., needs further research. In our study, we found that most curved electrodes occurred in larger lesions. Nevertheless, from a clinical perspective, these errors did not compromise SEEG recordings because the target was still located in the lesion and electrode bending did not result in complications, so we could still choose the appropriate electric contacts for thermocoagulation.

In addition, we also considered whether electrodes ran through different brain tissues, whether they touched cerebrospinal fluid, the closest distance from the ventricle, different entry points, skull thickness, extracranial soft tissue thickness, incidence angle and other related factors, but no statistical significance was found.

Besides the results of the above reports on the electrode trajectory, the factors that may affect the electrode implantation error also include the registration scanning mode, registration accuracy and machine performance (26, 36, 38). Different institutions register in different ways to identify the actual location of the patient’s head, mainly including bone and surface markers. Bone markers are generally considered to be more precise than surface markers (19, 33).

Recently, advances in magnetic resonance thermography, real-time thermal imaging and feedback control, are rapidly making LITT a practical solution in neurosurgery. And HH is the most common indication of MR-guided laser interstitial thermal therapy (MRgLITT) in pediatric epilepsy, studies have reported safety and efficacy of MRgLITT (13, 39–42). The accuracy of trajectory is equally important in the application of LITT, and similar to SEEG electrode implantation, LITT catheter can also be navigated implanted through framed or frameless systems. The difference is that LITT cannula is thicker than SEEG electrode (1.9 mm vs. 0.8 mm) due to the presence of cooling tube, thus requiring a larger safety threshold (13). Given the larger extent of LITT ablation, only one fiber is often required in the treatment of HH, which will facilitate the choice of trajectory. In conclusion, given the similar implantation principles, this study also has reference significance for the design of the LITT trajectory.

### 4.2. Seizure outcome

In the present study, of 30 procedures, 27 (90%) were seizure-free during follow-up. The mean follow-up duration was 31 months. This result is slightly better than those of other reports of HH treatment with SEEG-RFTC (11, 43). This may be due to our small sample size. In conclusion, the epilepsy remission rate of RFTC in HH is significantly better than that of traditional craniotomy (58%) (10), and it has good therapeutic effects in seizure recurrence patients with poor postoperative efficacy (7, 9, 23).

### 4.3. Complications

SEEG-RFTC has been reported to cause multiple complications in the treatment of HHs (11, 25). First of all, in terms of SEEG electrode implantation, intracranial hemorrhage and infection are the most common complications, and most complications are asymptomatic bleeding and do not require special treatment (25–27). For complications caused by electrode implantation, detailed intracranial vascular imaging needs to be performed before surgery and careful preoperative electrode planning is required to avoid damage to the vessels. At the same time, the higher the accuracy of electrode implantation, the lower the incidence of vascular damage. In addition to the complications related to electrode implantation, thermocoagulation of the target may also cause complications; for HH adjacent to the hypothalamus or the optic chiasm, thermocoagulation may cause complications in the surrounding tissues (11). In our study, one patient had short-term memory impairment. Another patient developed oculomotor palsy, which manifested as ptosis, which completely resolved 3 months after surgery. For SEEG-RFTC in the treatment of HHs in children, in order to avoid complications caused by thermocoagulation, an appropriate thermocoagulation target should be selected. The lesion produced by the procedure between two adjacent contacts is an ovoid with a length of approximately 6 mm and a maximal thickness of 3.5 mm (44), and the thermocoagulated tissue may to a certain degree resemble edema. When selecting the thermocoagulation target, an appropriate distance from the surrounding important tissues should be maintained. Overall, compared with traditional craniotomy, endoscopy, and radiosurgery for HH, SEEG-RFTC is associated with significantly fewer and less severe complications (7, 8, 11, 24). SEEG-RFTC is a safe and effective treatment modality for HH.

### 4.4. Limitations

The retrospective nature of the present study is the most important limitation. The small number of patients with patent cranial sutures and mild differences in skull angle and thickness may have influenced the electrode implantation error. Moreover, the literature review in this article is descriptive only.

## 5. Conclusion

Robot-assisted SEEG-RFTC is a safe, effective and accurate method for HH treatment that is gaining popularity. Explanatory variables significantly associated with TPLE in multivariate analysis include whether the intracranial electrode is bent, the intracranial electrode length and the entry point error. The influence of these factors on the target error should be considered in the design of electrode trajectories. Since the basis of the implantation methodology is the same, we believe that these findings also apply to MRIgLITT for the treatment of HH.

## Data availability statement

The raw data supporting the conclusions of this article will be made available by the authors, without undue reservation.

## Ethics statement

Ethical review and approval was not required for the study on human participants in accordance with the local legislation and institutional requirements. Written informed consent from the patients/participants or patients/participants' legal guardian/next of kin was not required to participate in this study in accordance with the national legislation and the institutional requirements.

## Author contributions

PL: Data curation, Formal analysis, Investigation, Writing – original draft, Conceptualization, Visualization. YZ: Conceptualization, Data curation, Formal analysis, Investigation, Visualization, Writing – original draft. QZ: Data curation, Investigation, Writing – original draft. YY: Data curation, Investigation, Writing – original draft. MW: Data curation, Formal analysis, Writing – original draft. ReZ: Data curation, Formal analysis, Writing – original draft. HL: Conceptualization, Methodology, Writing – review & editing. SG: Methodology, Project administration, Supervision, Writing – review & editing. RuZ: Funding acquisition, Methodology, Project administration, Supervision.
